# Supplementary material for: Synthesis of Novel Polymer-Assisted Organic-Inorganic Hybrid Nanoflowers and Their Application in Cascade Biocatalysis
Source: Molecules. 2023 Jan 14;28(2):839. doi: 10.3390/molecules28020839 (PMC9864776; doi:10.3390/molecules28020839)
Supplement: Supplementary file 1 [file molecules-28-00839-s001.zip › molecules-2028754-supplementary.pdf]

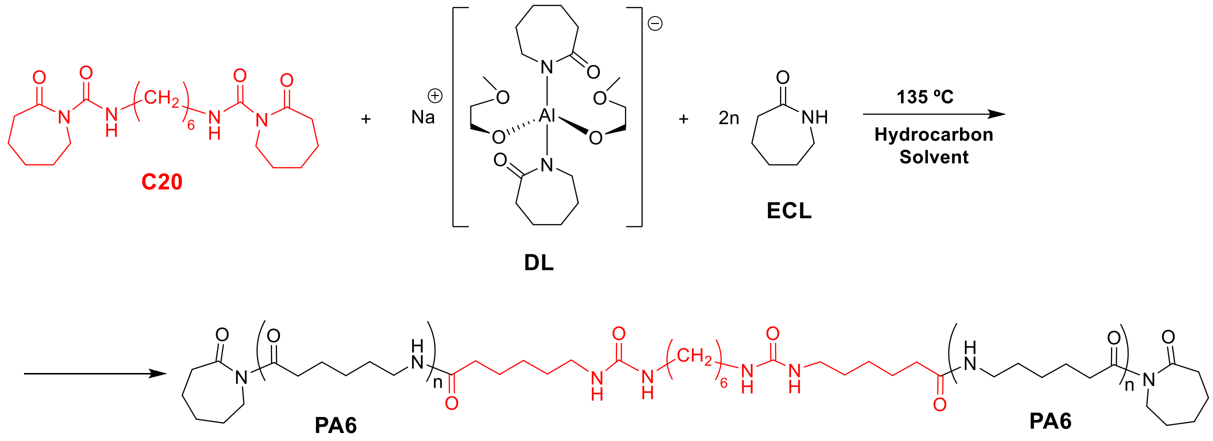

Figure S1. Chemical reactions occurring during AAROP of ε-caprolactam to PA6 MP. The chemical structure of the C20 imide activator and of the AAROP initiator dicaprolactamato-bis-(2-methoxyethoxo)-aluminate (DL) are also opresented.

Table S1. Elemental analysis of PANF samples by SEM/EDX with ZAF corrections.

	Sample denomination							
	NF GH/PA		NF GH&PA		NF G/PAiH		NF G&PAiH	
Element, wt.%	ZNF	ZPA6	ZNF	ZPA6	ZNF	ZPA6	ZNF	ZPA6
Cu K	32.2	2.5	35.5	2.3	30.4	0.0	27.4	2.5
P K	11.5	1.2	11.6	1.3	9.5	1.4	9.3	1.8

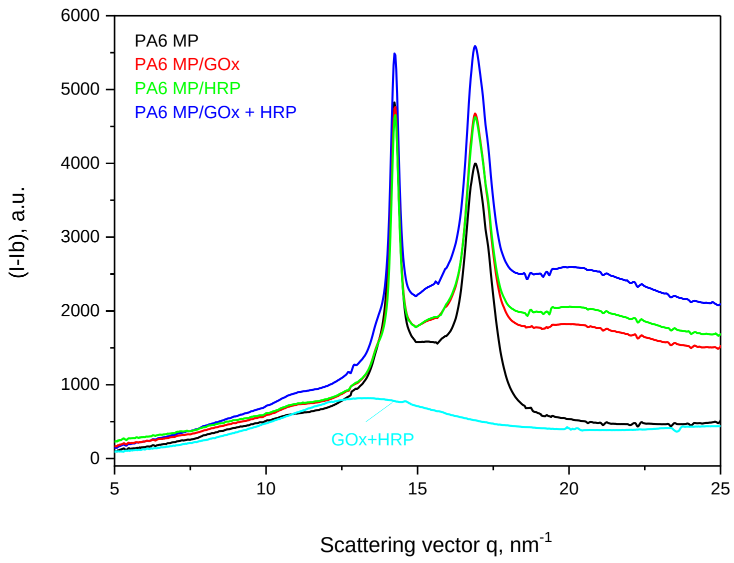

Figure S2. WAXS linear profiles of PA6 MP support before and after adsorption immobilization of GOx, HRP or GOx+HRP. The diffuse amorphous peak of the GOx+HRP mixture is also presented.

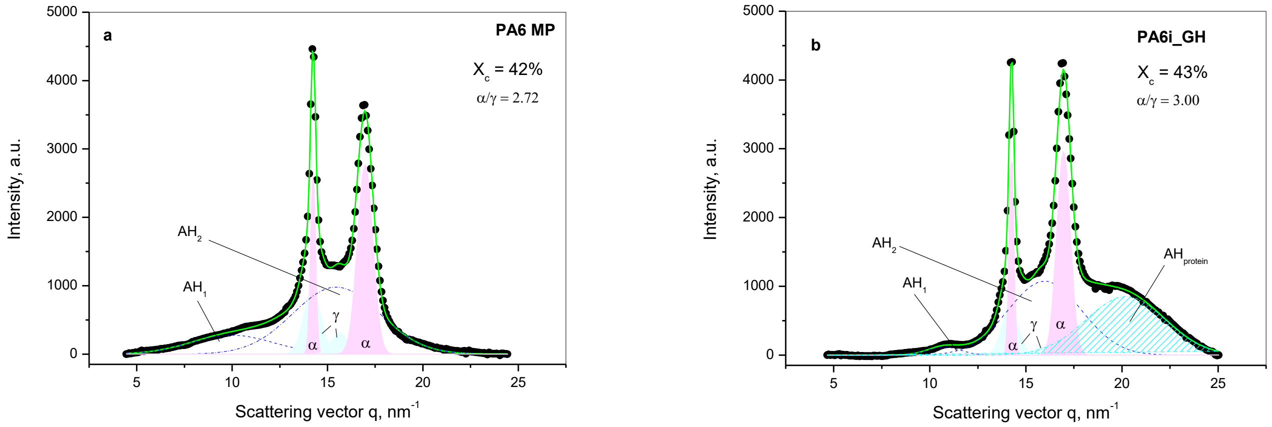

Figure S3. Deconvolution of the WAXS linear profiles of PA6 MP support before (**a**) and after (**b**) adsorption immobilization of GOx+HRP dyad. The new amorphous $AH_{protein}$ peak at q = 20.2 nm^{-1} found in the latter case is due to the H-bond formation of the absorbed enzymes with the support amorphous phase. Comparing the srystalline structure of PA6 MP and PA6_GH shows no changes in the support crystalline structure after enzyme absorption immobilization: the crystallinity index X_c and the α/γ polymorph relation remain quite similar, the differences being in the margen of the experimental error of the WAXS method.

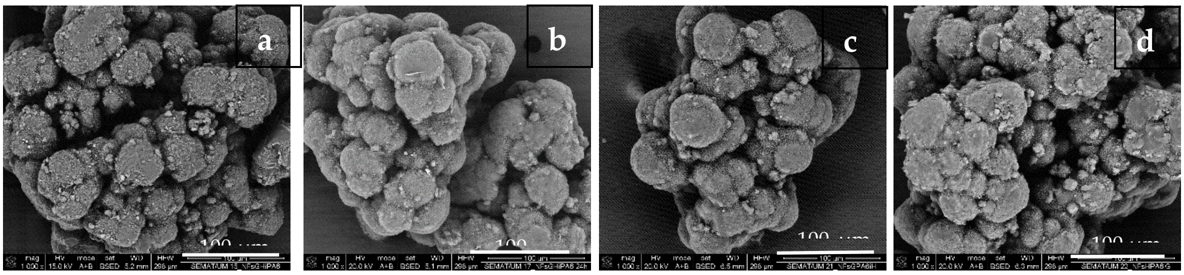

Figure S4. SEM micrographs of polymer-assisted hybrid nanoflowers (PANF) after the 6th catalytic cycle:.(**a**) - NF GH/PA; (**b**) - NF GH&PA; (**c**) NF G/PAiH; (**d**) NG G&PAiH. The brighter dot areas contain residual NaCl from the PBS or $Cu_3(PO_4)_2$ from the NF. For more details, see the main text.
